# Supplementary material for: Biocontrol efficacy of Bacillus licheniformis and Bacillus amyloliquefaciens against rice pathogens
Source: PeerJ. 2025 Jan 29;13:e18920. doi: 10.7717/peerj.18920 (PMC11786712; doi:10.7717/peerj.18920)
Supplement: Supplemental Information 1 [file peerj-13-18920-s001.docx]

**Table S1.** Colony morphology, cell morphology and Gram staining of all the rice root-associated bacteria

| **Isolates** | **Colony morphology** | **Cell morphology** | **Gram’s staining** |  | **Isolates** | **Colony morphology** | **Cell morphology** | **Gram’s staining** |
| --- | --- | --- | --- | --- | --- | --- | --- | --- |
| FE1 | Off white, Round, Medium, Smooth, Transparent | Large rods | -ve |  | LR4 | White, Round, Large, Concave, Opaque | Large rods | +ve |
| FE2 | White, Irregular, Small, Smooth, Shiny | Very small rods | +ve |  | LR5 | White, Round, Small, Convex, Dull | Very small rods | +ve |
| FE3 | Yellow, Round, Medium, Smooth, Shiny | Small rods | -ve |  | LR6 | White, Irregular, Large, Smooth, Opaque | Very small rods | +ve |
| FE4 | Off white, Round, Small, Smooth, Transparent | Very small rods | +ve |  | LR7 | Off white, Irregular, Large, Concave, Dull | Very small rods | +ve |
| FE5 | Brown, Round, Small, Smooth, Opaque | Medium rods | -ve |  | LR8 | White, Circular, Medium, Concave, Dull | Large rods | +ve |
| FE6 | Off white, Irregular, Medium, Concave, Shiny | Small rods | -ve |  | LR9 | Off white, Circular, Large, Concave, Shiny | Medium rods | -ve |
| FE7 | Brown, Round, Large, Smooth, Shiny | Small rods | +ve |  | LR10 | White, Circular, Medium, Smooth, Concave | Medium rods | -ve |
| FE8 | Off white, Round, Small, Smooth, Dull | Medium rods | +ve |  | LR11 | Off white, Circular, Medium, Smooth, Convex | Medium rods | +ve |
| FE9 | Brown, Round, Medium, Smooth, Shiny | Very small rods | -ve |  | LR12 | White, Circular, Medium, Convex, Convex | Medium rods | -ve |
| FE10 | Yellow, Round, Medium, Smooth, Transparent | Very small rods | -ve |  | LR13 | Off white, Circular, Small, Convex, Centered | Small rods | +ve |
| FE11 | White, Irregular, Large, Convex, Transparent | Small rods | +ve |  | LR14 | Pink, Circular, Medium, Smooth, Concave | Small rods | -ve |
| FR1 | Dark brown, Round, Large, Concave, Transparent | Medium rods | -ve |  | LR15 | Off white, Circular, Medium, Concave, Convex | Very small rods | -ve |
| FR2 | Off white, Irregular, Small, Convex, Opaque | Very small rods | -ve |  | LR16 | Off white, Circular, Large, umbonate, Elevated | Small rods | +ve |
| FR3 | Light brown, Round, Small, Smooth, Transparent | Medium rods | +ve |  | LR17 | Pink, Irregular, Medium, Concave, Scanty | Medium rods | +ve |
| FR4 | Brown, Irregular, Medium, Convex, dull | Very small rods | -ve |  | LR18 | White, Circular, Medium, Concave, Shiny | Small rods | +ve |
| FR5 | Off white, Round, Small, Smooth, Flowers | Very small rods | -ve |  | LR19 | Light pink, Circular, Large, Umbonate, Centered | Large rods | -ve |
| FR6 | Off white, Irregular, Small, Curved, Transparent | Medium rods | +ve |  | LR20 | Light yellow, Circular, Medium, Smooth, Dull | Very small rods | +ve |
| FR7 | White, Irregular, Medium, Concave, Opaque | Medium rods | -ve |  | LR21 | Off white, Circular,Medium, Concave, Scanty | Small rods | +ve |
| FR8 | Off white, Irregular, Medium, Concave, Dull | Small rods | -ve |  | LR22 | White, Irregular, Medium, Convex, Flower like | Medium rods | +ve |
| FR9 | Off white, Round, Small, Concave, Shiny | Very small rods | +ve |  | LR25 | Light yellow, Irregular, Medium, Smooth, Hairy | Small rods | +ve |
| FR10 | Dark brown, Round, Small, Concave, Dull | Very small rods | +ve |  | LR26 | White, Round, Medium, Elevated, White lines | Very small rods | +ve |
| FR11 | Dark brown, Round, Medium, Concave, Shiny | Very small rods | -ve |  | LR27 | Off white, Rod, Small, Smooth, Rod | Very small rods | -ve |
| FR12 | Light brown, Irregular, Medium, Convex, Dull | Large rods | +ve |  | LR28 | Off white, Irregular, Large, Smooth, Jelly like | Very small rods | -ve |
| FR13 | Off white, Round, Large, Smooth, Transparent | Medium rods | +ve |  | LR29 | Off white, Regular, Medium, Concave, Shiny | Small rods | -ve |
| FR14 | Off white, Round, Small, Smooth, Transparent | Very small rods | -ve |  | LR30 | Yellow, round, Small, Smooth, Normal | Small rods | -ve |
| FR15 | Off white, Round, Medium, Concave, Transparent | Large rods | +ve |  | LR31 | Off white, Irregular, Medium, Smooth, Scanty | Small rods | -ve |
| FR16 | Yellow, Irregular, Small, Smooth, opaque | Very small rods | -ve |  | LR32 | Off white, Irregular, Medium, Convex, Scanty | Very small rods | -ve |
| FR17 | Light brown, Round, Small, Smooth, Shiny | Very small rods | +ve |  | LR33 | Light pink, Circular, Small, Convex, Normal | Medium rods | +ve |
| LE1 | White, Circular, Medium, Concave, Scanty | Small rods | -ve |  | LR34 | Pink, rod, Large, Concave, Smooth | Large rods | +ve |
| LE2 | Pink, Round, Large, Convex, Scanty | Large rods | -ve |  | LR35 | Yellow, Circular, Medium, Smooth, Convex | Medium rods | +ve |
| LE3 | Yellow, Circular, Small, Smooth, Concave | Large rods | -ve |  | LR36 | Pink, Circular, Medium, Smooth, Normal | Medium rods | -ve |
| LE4 | White, Circular, Medium, Smooth, Concave | Medium rods | +ve |  | PE1 | Off white, Irregular, Small, Convex, Transparent | Large rods | +ve |
| LE5 | Off white, Circular, Medium, Smooth, Scanty | Medium rods | +ve |  | PE2 | White, Circular, Small, Smooth, Transparent | Small rods | -ve |
| LE6 | Cream, Circular, Medium, Smooth, Normal | Medium rods | -ve |  | PE3 | Off white, Circular, Medium, Concave, Shiny | Small rods | -ve |
| LE7 | Cream, Rod, Small, Flat, Normal | Small rods | +ve |  | PE4 | Brown, Circular, Medium, Smooth, Transparent | Very small rods | +ve |
| LE8 | Off white, Irregular, Large, Convex, Scanty | Medium rods | +ve |  | PE5 | Light brown, Irregular, Medium, Smooth, Wavy edges | Medium rods | -ve |
| LE9 | White, Irregular, Large, Concave, Scanty | Medium rods | -ve |  | PE6 | Off white, Circular, Medium, Smooth, Wavy | Small rods | +ve |
| LE10 | Pink, Irregular, Medium, Convex, Scanty | Very small rods | -ve |  | PE7 | Pink, Irregular, Medium, Convex, Wavy | Small rods | +ve |
| LE11 | Off white, Irregular, Medium, Smooth, Net like | Very small rods | +ve |  | PR1 | Off white, Round, Large, Umbonate, Transparent | Medium rods | +ve |
| LE12 | Off white, Circular, Small, Convex, Smooth | Small rods | -ve |  | PR2 | Light brown, Circular, Medium, Convex, Irregular | Very small rods | +ve |
| LE13 | Pink, Circular, Small, Smooth, Porous like | Small rods | -ve |  | PR3 | Off white, Irregular, Small, Convex, Irregular | Very small rods | +ve |
| LE14 | Off white, rod, Medium, Convex, Scanty | Large rods | +ve |  | PR4 | Off white, Irregular, Small, Smooth, Transparent | Small rods | -ve |
| LE15 | Off white, Irregular, Medium, Convex, Shiny | Small rods | -ve |  | PR5 | Off white, Round, Medium, Concave, Irregular | Medium rods | -ve |
| LE16 | Off white, Irregular, Large, Smooth, Rough | Very small rods | +ve |  | PR6 | Light cream, Circular, Medium, Smooth, Shiny | Medium rods | +ve |
| LE17 | Off white, Circular, Small, Smooth, Granular | Small rods | +ve |  | PR7 | Light cream, Circular, Medium, Smooth, Shiny | Small rods | +ve |
| LR1 | Off white, Round, Large, Smooth, Transparent | Medium rods | +ve |  | PR8 | White, Circular, Small, Convex, Dull | Very small rods | -ve |
| LR2 | Brown, Circular, Large, Smooth, Transparent | Small rods | -ve |  | PR9 | Off white, Irregular, Medium, smooth, Dull | Small rods | -ve |
| LR3 | Light pink, Round, Large, Convex, Opaque | Medium rods | -ve |  | PR10 | Light brown, Irregular, Medium, Convex, Shiny | Small rods | -ve |
